# Mental Health in Athletes: Where Are the Treatment Studies?

**DOI:** 10.3389/fpsyg.2022.781177

**Published:** 2022-07-04

**Authors:** Rebecka Ekelund, Stefan Holmström, Andreas Stenling

**Affiliations:** ^1^Department of Psychology, Umeå University, Umeå, Sweden; ^2^Umeå School of Sport Sciences, Umeå University, Umeå, Sweden; ^3^Department of Sport Science and Physical Education, University of Agder, Kristiansand, Norway

**Keywords:** mental disorders, mental health problems, interventions, psychotherapy, sports

## Abstract

In recent years, athletes’ mental health has gained interest among researchers, sport practitioners, and the media. However, the field of sport psychology lacks empirical evidence on the effectiveness of psychotherapeutic interventions for mental health problems and disorders in athletes. Thus far, intervention research in sport psychology has mainly focused on performance enhancement using between-subject designs and healthy athlete samples. In the current paper, we highlight three interrelated key issues in relation to treating mental health problems and disorders in athletes. (i) How are mental health and mental health problems and disorders defined in the sport psychology literature? (ii) How are prevalence rates of mental health problems and disorders in athletes determined? (iii) What is known about psychotherapeutic interventions for mental health problems and disorders in athletes? We conclude that the reliance on different definitions and assessments of mental health problems and disorders contributes to heterogeneous prevalence rates. In turn, this limits our understanding of the extent of mental health problems and disorders in athletes. Furthermore, knowledge of the effectiveness of psychotherapeutic interventions for athletes with mental health problems and disorders is scarce. Future research should include athletes with established mental health problems and disorders in intervention studies. We also propose an increased use of N-of-1 trials to enhance the knowledge of effective psychotherapeutic interventions in this population.

## Introduction

There has been an increasing interest in athletes’ mental health among researchers and sport practitioners in the past decade. Several reviews and position statements have recently been published on this issue (e.g., Reardon et al., 2019; Kuettel and Larsen, 2020). Several top athletes also highlighted mental health problems and how it affected their sport performance during the recent Olympic Games in Tokyo, which major news outlets have reported on, such as the *New York Times* (Longman, 2021) and the *Washington Post* (Svrluga, 2021). Despite this increased interest, studies on the effectiveness of psychotherapeutic interventions for mental health problems and disorders in athletes are almost nonexistent (Stillman et al., 2019). Most previous research has focused on interventions for performance enhancement (e.g., Schenk and Miltenberger, 2019), resulting in an apparent knowledge gap that needs to be addressed in future research.

Researchers have argued that athletes differ from the general population (e.g., Reardon et al., 2019) in, but not limited to, personality traits (e.g., narcissistic tendencies, perfectionism, and competitiveness), behaviors (e.g., risk-taking and superstitious rituals; Stillman et al., 2016), and barriers to help-seeking (Gulliver et al., 2012). Furthermore, some have argued that these differences, among others, need to be considered when working in a psychotherapeutic setting with athletes (Stillman and Farmer, 2021). However, empirical evidence is scarce, and research is needed to examine whether, and if so, how athletes differ from the general population (Gouttebarge et al., 2019). In addition, researchers have put much effort into determining prevalence rates of mental health problems and disorders in athletes. However, there is an extreme heterogeneity of prevalence rates in the published literature, making it difficult to determine the extent of mental health problems and disorders in athletes (Gouttebarge et al., 2019).

In light of the above, in the current paper, we will discuss three interrelated key issues related to research on treating mental health problems and disorders in athletes. (i) How are mental health and mental health problems and disorders defined in the sport psychology literature? This has important implications for both assessing the prevalence of and treating mental health problems and disorders. (ii) How are prevalence rates of mental health problems and disorder in athletes determined? (iii) What is known about psychotherapeutic interventions for mental health problems and disorders in athletes? We conclude by outlining several issues in need of further research related to treating mental health problems and disorders in athletes and suggest ways to advance knowledge in this area.

## Mental Health in Sport Psychology

### Definitions of Mental Health, Mental Health Problems, and Mental Disorders

Historically, mental health has been defined in many different ways and how to define it is still under debate (e.g., Keyes, 2002, 2005; Galderisi et al., 2015). More recently, a narrative review on mental health in athletes by Lundqvist and Andersson (2021) concluded that what is considered mental health or mental health problems will vary depending on three factors: definition, theoretical perspective, and the assessment chosen by researchers. Hence, an essential prerequisite when researching mental health is to provide a clear definition and a well-grounded theoretical perspective of mental health and mental health problems (Lundqvist and Andersson, 2021).

Several researchers in sport psychology have suggested conceptualizing mental health as part of a continuum rather than adhering to strict diagnostic criteria and viewing it as a binary state (Moore and Bonagura, 2017; Rice et al., 2021). On the other hand, Lundqvist and Andersson (2021) argue that continuum models do not provide any guidance regarding how to interpret symptoms and whether symptoms should be viewed as natural reactions to sports or early signs of mental health problems or disorders. Many elite athletes are expected over time to move back and forth along the continuum without necessarily being at risk of developing clinically relevant mental health problems or needing treatment (Lundqvist and Andersson, 2021). Thus, caution is needed not to pathologize everyday human experiences (Henriksen et al., 2020). Despite this increased interest and recent efforts, Lundqvist and Andersson (2021) argue that it is unlikely that consensus will be reached on a uniform definition of mental health in elite sport contexts.

The lack of a uniform definition in the sport psychology literature is also evident in relation to mental health problems and disorders. The terms mental health problem and mental disorder are sometimes used interchangeably despite referring to different levels of severity and diagnosis. A mental disorder refers to a specific psychiatric diagnosis based on several criteria in the *Diagnostic and Statistical Manual of Mental Disorders* (*DSM*-5; American Psychiatric Association, 2013), whereas mental health problems usually refer to subclinical psychological ill-being without necessarily fulfilling clinical criteria according to the *DSM*-5. Symptoms of subclinical psychological ill-being are often signs of how mental health can fluctuate without developing into an all-encompassing disorder (i.e., cognitive and emotional disturbance, abnormal behaviors, and/or impaired functioning; American Psychiatric Association, 2013). An example from the sport context is performance anxiety versus an established psychiatric anxiety disorder (e.g., generalized anxiety disorder, social anxiety, and obsessive–compulsive disorder). Performance anxiety most often occurs before a performance and is a passing state, whereas generalized anxiety disorder, for example, is an ongoing state leading to impaired functioning in different areas of life (Reardon et al., 2021).

Although these can be viewed as subtle differences, the nuances have important practical implications related to assessment and treatment. Disregarding these subtle differences increases the risk of underestimating or overestimating mental health problems and disorders in athletes. Relying on different definitions and operationalizations increases the heterogeneity of prevalence rates in the published literature, which, in turn, creates confusion and uncertainty rather than clarity in terms of the extent of mental health problems and disorders in athletes.

### Prevalence of Mental Health Problems and Disorders in Athletes

Prevalence rates of mental health problems and disorders in athletes are often studied with quantitative and cross-sectional methods using self-reported data *via* questionnaires (Kuettel and Larsen, 2020). Consequently, prevalence rates of mental health problems and disorders among athletes vary and are more prominent in some samples than others, often dependent on the type of study design and type of assessment. Prevalence rates are generally higher in studies adopting a broader definition of mental health problems and self-report measures compared to studies that limit assessment to psychiatric diagnosis and clinical evaluation. For example, prevalence rates for depression in athletes range from 4% when clinically assessed (Schaal et al., 2011) to 48% when self-reported (Foskett and Longstaff, 2018), whereas rates for anxiety varies from 9% when clinically assessed (Schaal et al., 2011) to 16% when self-reported (Åkesdotter et al., 2020). Åkesdotter et al. (2020) included both psychiatric disorders and psychological distress symptoms in their definition of mental health problems and used self-report measures, whereas Schaal et al. (2011) examined the prevalence rates of mental health problems based on psychological disorders found in the *DSM-IV* or International Classification of Diseases 10th version (*ICD*-10) with a licensed caregiver conducting additional clinical evaluations.

These discrepancies show that prevalence rates of mental health problems and disorders differ substantially based on the definition and operationalization of mental health problems (e.g., psychiatric diagnosis versus symptoms of psychological distress), type of assessment (e.g., self-report versus clinically assessed), and instruments used, and contribute to the heterogeneity of prevalence rates for mental health problems and disorders in athletes. This heterogeneity and lack of consensus regarding how to assess prevalence limits the understanding of athletes’ mental health problems and disorders. Furthermore, Gouttebarge et al. (2019) argued that prevalence rates for current elite athletes might be slightly higher than in the general population; however, comparisons were not possible due to the lack of reference group from the general population in the studies included in their meta-analysis. Nevertheless, despite these methodological issues, the available evidence indicates that the prevalence rates of the most common mental health problems and disorders seem comparable with those of the general population (Gorczynski et al., 2017; Moesch et al., 2018).

## Psychotherapeutic Interventions in Sport Psychology

Many recommendations have been put forward about how to address mental health problems and disorders in sport contexts, such as prevention strategies (Reardon et al., 2019), mental health officers (Henriksen et al., 2020), and new screening tools to detect symptoms of mental health problems (Gouttebarge et al., 2021). Surprisingly, very few of these recommendations include calls for more rigorous and controlled studies on psychotherapeutic interventions for athletes. Intervention research has mainly focused on performance enhancement (e.g., Sappington and Longshore, 2015; Schenk and Miltenberger, 2019), although the constant pressure to perform may increase athletes’ vulnerability to mental health problems (Kuettel and Larsen, 2020). The scarcity of research on interventions for athletes’ mental health problems and disorders has resulted in a critical knowledge gap related to the effectiveness of interventions (Stillman et al., 2019) and the underlying mechanisms that account for intervention outcomes (Gross et al., 2016).

Cognitive behavioral therapy (CBT) has been recommended as an “excellent choice” for treating athletes with mental health problems or disorders because it involves procedures that athletes commonly use, such as structure, direction, goal setting, and practice (Stillman et al., 2016). However, to our knowledge, there are no studies conducted on the effectiveness of CBT on athletes with established mental health problems or disorders. Nevertheless, given that CBT is a well-researched form of psychotherapy and established as one of the most effective and common treatments for a wide range of mental health problems and disorders (Hofmann et al., 2012), it is understandable why CBT is recommended. Despite the lack of clinical studies using CBT for mental health problems or disorders in athlete populations, a limited number of case studies have been conducted using CBT principles with athletes. For example, Gustafsson et al. (2017) performed a six-session exposure intervention with a 17-year-old cross-country skier experiencing a high level of performance anxiety. Furthermore, McArdle and Moore (2012) describe how one of the authors employed key CBT principles when working with a 26-year-old rugby player with a dysfunctional perfectionist mindset. Participants in the abovementioned studies were not diagnosed with a clinical diagnosis but were included on the basis of experiencing mental health problems and underperforming. However, Lundqvist (2020) provides an example of how to use behavioral activation when working with a former Olympic athlete who developed depression (according to the Montgomery-Åsberg Depression Rating Scale) after retirement.

In addition to CBT, so-called third-wave behavioral therapies such as acceptance and commitment therapy (ACT) and compassion-focused therapy (CFT) seem promising (e.g., Ruiz, 2010; Craig et al., 2020) and should also be evaluated in athlete populations. ACT stems from the traditional behavior and cognitive therapies, such as CBT, but with a stronger emphasis on mindfulness and acceptance (Hayes et al., 2006). ACT has been widely researched in clinical samples with strong evidence for a wide range of mental health problems (Ruiz, 2010), such as anxiety (Swain et al., 2013), depression (Bai et al., 2020), and chronic pain (Veehof et al., 2016). Since introducing ACT (Hayes et al., 1999), many interventions in sport psychology have drawn from the ACT model and its six core processes (i.e., values, contact with the present moment, committed action, acceptance, self as a context, and defusion). Sport psychology researchers have mostly adopted the parts about being present in the moment and accepting internal events (i.e., thoughts and emotions) to enhance performance (e.g., The Mindfulness-Acceptance-Commitment approach; Moore, 2009). Mindfulness-based interventions for enhanced athletic performance show promising results of being effective in improving characteristics associated with well-being, such as psychological flexibility and anxiety (Sappington and Longshore, 2015). However, the field currently lacks clinical intervention studies testing the ACT model as a psychotherapeutic intervention in athletes with mental health problems or disorders.

A small number of intervention studies in sport contexts have included parts of the ACT model (see Lundgren et al., 2020; Moesch et al., 2020). However, in these studies, participants were recruited based on characteristics related to their sport participation (e.g., current injury, motivation to participate), not that they explicitly needed treatment for mental health problems or disorders. It is difficult to draw conclusions about the effectiveness of an intervention on mental health problems (e.g., anxiety, depression, and psychological rigidity) based on research with healthy samples. Research on compassion-based interventions (Neff, 2003; Gilbert, 2009) in sport is also scarce but is gaining interest (Craig et al., 2020). However, despite an increased interest, a scoping review (Röthlin, 2019) on the role of self-compassion in competitive sport settings only found one intervention study (i.e., Mosewich et al., 2013). Given the lack of empirical evidence, athletes experiencing mental health problems or disorders need to be included in future studies to evaluate the effectiveness of interventions based on CBT, ACT, or CFT.

### Targeted and Disorder-Specific or Transdiagnostic Treatment?

There have also been calls for developing comprehensive, targeted, and disorder-specific treatment models for athletes (e.g., Rice et al., 2016). However, this suggestion is problematic for several reasons. First, to adopt a targeted, disorder-specific treatment with an individual, that person must fulfill the criteria for a specific disorder, and only those criteria. This is rarely the case and comorbidity (i.e., the occurrence of two or more psychiatric disorders simultaneously) is more often the rule than the exception (Krueger and Eaton, 2015). Second, a sole focus on those with confirmed disorders will exclude many athletes struggling with subclinical mental health problems (Reardon et al., 2019). Third, how can we develop new, comprehensive, targeted, and disorder-specific treatment models when there is a lack of evidence related to the effectiveness of already established psychotherapeutic treatment models (e.g., CBT, ACT, and CFT) in athletic samples?

Researchers in the field of sport psychology seem to agree that mental health problems are more than just specific disorders and that the full range of mental health problems need to be considered (e.g., Moesch et al., 2018; Henriksen et al., 2020; Kuettel and Larsen, 2020; Lundqvist and Andersson, 2021). In addition, due to issues such as categorical overlap and high comorbidity rates (Meidlinger and Hope, 2017; Reardon, 2017), recognition is growing in terms of acknowledging that traditional psychiatric diagnoses are flawed due to the limitations (e.g., topographical approach, syndromal classification, and diagnosis overlap) of the current *DSM*-5 diagnostic system, and thus the treatments of them. Because of this, the field of clinical psychology is advancing toward transdiagnostic approaches aimed at targeting underlying mechanisms (e.g., emotional and cognitive avoidance, attentional focus, and worry) hypothesized to drive and maintain a person’s mental health problems (Frank and McKay, 2020). The field of sport psychology would benefit from following this trend and research on how transdiagnostic approaches (e.g., ACT and CFT) can be used in interventions with athletes is warranted. Well-designed clinical studies evaluating established psychotherapeutic interventions in athletes should be prioritized over developing new, comprehensive, targeted, and disorder-specific treatment models.

## General Discussion

When examining the literature on the prevalence rates of mental health problems and disorders in athletes, it is apparent that the field lacks a shared language to discuss mental health, mental health problems, and mental disorders, and the terms are often not clearly defined. The lack of consensus regarding the definition of mental health and mental health problems likely contributes to heterogeneous prevalence rates for mental health problems and disorders among athletes (Lundqvist and Andersson, 2021). Furthermore, using clinical cutoff values and diagnostic criteria are essential in future research; however, a strict focus on cutoff values and criteria is not very helpful when designing psychotherapeutic interventions for athletes with mental health problems and disorders. In a treatment setting, it is likely more effective to focus on factors underlying and underpinning mental health problems rather than fulfilling diagnostic criteria (Hayes et al., 2020).

In line with the increasing interest in research on mental health problems and disorders in sport, it is reasonable to assume that research on treatments for such problems would follow. However, this has not been the case. Clinical studies testing well-researched and evidence-based psychotherapeutic interventions in athletes with mental health problems or disorders are long overdue within sport psychology. Consequently, interventions in sport psychology have been criticized to be “a shot in the dark” (Moore and Bonagura, 2017, p. 178), and researchers have expressed that athletes deserve to receive support for their mental health equal to what they receive for their physical health (Currie et al., 2021).

We argue that an increased use of N-of-1 studies and including athletes with established mental health problems or disorders in intervention studies would greatly benefit the understanding of effective treatments. N-of-1 studies have been recommended in contexts where variability in patient response is large, when the evidence is limited, and/or when the patient differs in important ways from the population participating in conventional randomized trials (Mirza et al., 2017). Furthermore, an N-of-1 approach is especially valuable when taking on new research areas (Barker et al., 2020). All these recommendations are applicable to research on interventions for mental health problems and disorders in athletes where the variability in type and prevalence varies greatly (e.g., Rice et al., 2016; Gouttebarge et al., 2019), and the evidence for interventions for mental health problems and disorders is scarce (Stillman et al., 2019). Furthermore, the athletic population has been suggested to differ from the general population in how mental health problems and disorders are expressed and factors that affect mental health, which may impact the effectiveness of interventions in this population (Reardon et al., 2019). However, this suggestion requires confirmation in empirical studies.

### Summarizing Conclusion

There is an urgent need for well-designed clinical studies testing established psychotherapeutic interventions in athletes with established mental health problems or disorders. We argue that N-of-1 studies provide a promising approach to build a knowledge base for treating mental health problems and disorders in athletes, which would aid in psychologists’ mission to offer the best possible support for athletes who need it.

## Data Availability Statement

The original contributions presented in the study are included in the article/supplementary material, further inquiries can be directed to the corresponding author.

## Author Contributions

RE wrote the manuscript. AS and SH critically reviewed and revised for intellectual content before submission. The authors discussed and agreed upon the main messages during the paper’s preparation. All authors contributed to the article and approved the submitted version.

## Conflict of Interest

The authors declare that the research was conducted in the absence of any commercial or financial relationships that could be construed as a potential conflict of interest.

## Publisher’s Note

All claims expressed in this article are solely those of the authors and do not necessarily represent those of their affiliated organizations, or those of the publisher, the editors and the reviewers. Any product that may be evaluated in this article, or claim that may be made by its manufacturer, is not guaranteed or endorsed by the publisher.
